# MOT-Assisted Object-Level Point Cloud Extraction from Multi-View Observations

**DOI:** 10.3390/s26165032

**Published:** 2026-08-07

**Authors:** Cheng Ju, Zejing Zhao, Chaowen Shen, Xuantong Li, Akio Namiki

**Affiliations:** Graduate School of Engineering, Chiba University, 1-33 Yayoicho, Inage-ku, Chiba 263-8522, Japan; 21wd4101@student.gs.chiba-u.jp (C.J.); 24wd4101@student.gs.chiba-u.jp (Z.Z.); 24wd9102@student.gs.chiba-u.jp (C.S.); 24wm9102@student.gs.chiba-u.jp (X.L.)

**Keywords:** multi-object tracking, object-level point clouds, SLAM

## Abstract

Object-level point cloud extraction is relevant to robotic perception and may provide useful object-centric observations for mapping and SLAM. Many existing methods rely on per-frame instance segmentation, resulting in high computational cost and limited ability to extract multiple objects simultaneously in multi-object scenarios. This paper proposes an efficient MOT-assisted pipeline for multi-view object point cloud extraction. The pipeline first employs 2D multi-object tracking (MOT) to establish consistent object correspondences across views, and then combines monocular depth-based reconstruction with multi-view geometric association to estimate coarse object locations. An adaptive spherical proposal and a density-based refinement strategy are further introduced to extract clean object-specific point clouds while suppressing background noise and outliers. Experiments on the DTU, MVImgNet, and ScanNet++ datasets demonstrate the effectiveness of the proposed method. Relative to the unprocessed scene-level point cloud, the Chamfer Distance is reduced from 90.97 mm to 12.52 mm and Precision increases from 0.2998 to 0.8776 on DTU dataset Sequence 30. On MVImgNet, the Chamfer Distance decreases from 1.23 m to 0.46 m and Precision increases from 0.4777 to 0.9337, demonstrating effective removal of non-target scene points under real-world viewing conditions. Moreover, the proposed method reduces the cost of object-level association and provides competitive object-level extraction quality under the tested settings. Compared with the closely matched segmentation-based extraction, the proposed method provides lower measured object extraction runtime and built-in cross-view Track-ID association, while sacrificing some boundary accuracy.

## 1. Introduction

In recent years, the rapid development of robotics has significantly increased the demand for reliable perception and understanding of complex three-dimensional environments. Autonomous robots are now expected to operate in dynamic and unstructured scenes for applications such as inspection, logistics, service robotics, and autonomous navigation [1,2]. To achieve robust interaction with the physical world, robots must not only localize themselves accurately but also perceive surrounding objects in a structured and geometrically meaningful way [3,4]. In this context, point cloud representation has become a fundamental medium for robotic perception, as it preserves explicit 3D spatial information of the environment and supports downstream tasks such as mapping, scene understanding, object reconstruction, and manipulation. Consequently, point-cloud-based simultaneous localization and mapping (SLAM) has emerged as a key component in modern robotic systems, enabling robots to incrementally reconstruct the surrounding scene while estimating their own poses.

Despite the substantial progress of point cloud SLAM, extracting object-level point clouds from reconstructed scenes remains challenging, especially in multi-object environments. Existing approaches often rely on semantic or instance segmentation to isolate target objects from dense point clouds. Although effective in some scenarios, these methods typically require heavy per-frame prediction, expensive 3D processing, and large annotation costs, which limit their efficiency and simultaneous multi-object extraction capability in practical robotic systems. Moreover, when the primary goal is to obtain object-level point cloud trajectories or object-specific 3D observations, full segmentation may be unnecessary and computationally redundant. In contrast, multi-object tracking (MOT) provides a lightweight alternative by maintaining temporally consistent object identities across image sequences using 2D detections and motion association, without requiring pixel-level masks. This property makes MOT particularly attractive for robotic perception pipelines where efficiency and temporal consistency are both critical.

Motivated by this observation, we explore the use of MOT as an auxiliary module for object-level point-cloud extraction, with potential relevance to object-centric mapping and SLAM. Instead of performing costly segmentation for every frame, our method leverages tracked 2D object trajectories across multiple views to associate persistent cross-view identities of the same object over time, and then uses these track-ID-consistent view associations to guide the extraction of corresponding 3D point subsets from the reconstructed scene. In this way, MOT serves as an efficient bridge between image-space object dynamics and point-cloud-level object representation, thereby supporting efficient, track-ID-aware simultaneous multi-object extraction. This perspective opens a practical direction for integrating mature tracking techniques into 3D robotic perception and object-centric SLAM.

In summary, the main contributions of this work are as follows:

1. We propose an MOT-assisted object point cloud extraction pipeline that integrates 2D multi-object tracking with point-cloud-based scene reconstruction, providing an efficient alternative to segmentation-heavy object extraction pipelines in robotic perception.
2. We design a multi-view object association strategy that leverages tracked object trajectories and object-centered viewing rays to establish cross-view geometric consistency, enabling reliable localization of object-specific 3D regions within the reconstructed scene.
3. We introduce a coarse-to-fine point cloud extraction scheme, where a dynamically estimated object-centered spatial range is first constructed to narrow the candidate point set, followed by density-based 3D denoising to refine the final object point cloud.

Through this design, the proposed method enables efficient, track-ID-aware multi-object point cloud extraction while preserving temporal consistency and geometric accuracy, demonstrating the potential of MOT as a practical enhancement for object-centric point cloud perception in robotic systems.

## 2. Related Works

### 2.1. Point Cloud Generation from Multi-View Observations

**Figure 1:**
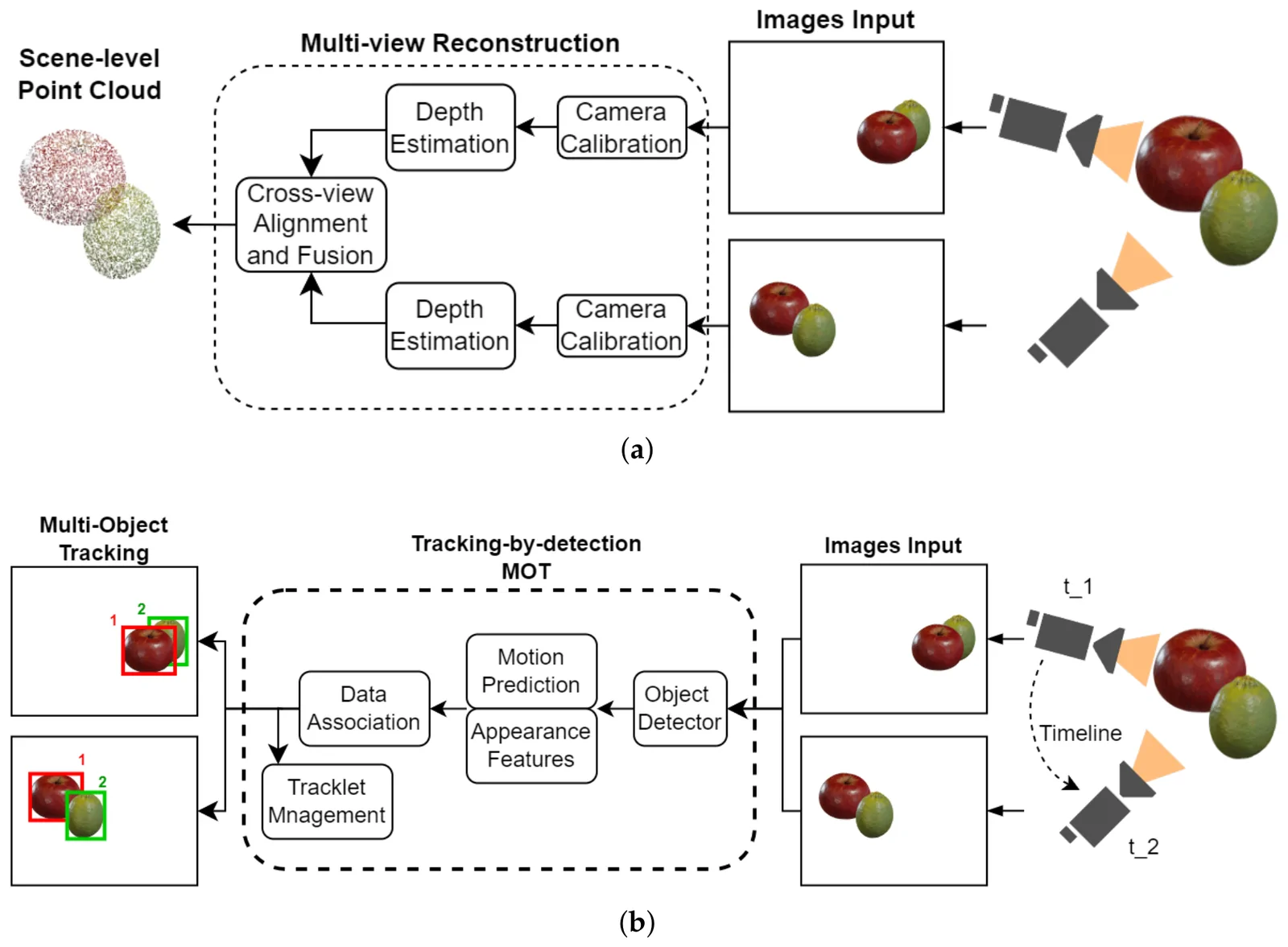
Conceptual illustrations of the background pipelines discussed in Section 2. (**a**) A generic multi-view reconstruction pipeline, in which geometry estimated from multiple calibrated observations is aligned and fused into a scene-level point cloud or mesh. (**b**) A typical tracking-by-detection MOT pipeline, in which frame-wise detections are associated using motion and appearance cues to produce persistent object identities and trajectories.

Recovering 3D geometry from multi-view observations has long been a core problem in computer vision and robotics. Traditional pipelines [5] typically rely on Structure-from-Motion (SfM) [6] and Multi-View Stereo (MVS) [7,8] to estimate camera poses and reconstruct scene geometry from image correspondences. These methods produce explicit point clouds or meshes and have been widely used in static scene reconstruction due to their geometric interpretability and compatibility with downstream tasks such as localization, mapping, and object-level analysis. However, their performance often degrades in challenging conditions such as textureless regions, repeated patterns, sparse viewpoints, or severe occlusions, where reliable feature matching and triangulation become difficult. A conceptual illustration is shown in Figure 1a.

To improve robustness and reconstruction quality, recent research has increasingly shifted toward learning-based novel view synthesis and 3D reconstruction methods. Neural radiance field (NeRF)-based approaches [9] model a scene as a continuous volumetric radiance field and achieve impressive rendering fidelity from posed images. Nevertheless, the implicit nature of NeRF makes geometry extraction relatively expensive, and both training and rendering are often computationally demanding for practical robotics pipelines. More recently, 3D Gaussian Splatting (3DGS) [10,11] and its variants have emerged as an efficient alternative by representing scenes with explicit Gaussian primitives, offering a favorable trade-off between reconstruction quality and rendering speed. This line of work has rapidly expanded from static novel-view synthesis to sparse-view reconstruction, dynamic scene modeling, and SLAM-oriented systems. Compared with classical point-based reconstruction, Gaussian-based representations can better preserve fine appearance details while still providing a bridge to explicit geometric representations such as point clouds.

Despite this progress, most existing point cloud generation pipelines remain primarily scene-centric: they aim to reconstruct the entire scene or a globally consistent representation, rather than directly isolating the point cloud of each target object. In robotic perception, however, many downstream tasks—such as manipulation, object-level mapping, or semantic scene understanding—require instance-level point cloud extraction instead of a monolithic scene reconstruction. A straightforward solution is to first reconstruct the whole scene and then perform 3D segmentation or instance separation, but this introduces extra computational cost and often depends on dense 3D annotations or strong geometric priors. Therefore, there remains a gap between general-purpose point cloud generation methods and efficient target-oriented point cloud extraction pipelines.

### 2.2. Multi-Object Tracking

Multi-object tracking (MOT) aims to maintain the identities and trajectories of multiple targets across image sequences. Modern 2D MOT systems are commonly built in a tracking-by-detection paradigm, where an object detector first produces frame-wise detections and a tracker then associates them over time. Early online methods such as SORT [12] formulate tracking mainly with a Kalman filter [13] and Hungarian matching, emphasizing real-time performance but relying heavily on detection quality and simple motion assumptions. Subsequent methods, exemplified by DeepSORT [14], augment motion cues with appearance embeddings to improve data association under occlusion and target interactions. A conceptual illustration is shown in Figure 1b.

More recent trackers have focused on improving association robustness while preserving efficiency. For example, ByteTrack [15] demonstrates that associating not only high-confidence detections but also low-confidence candidates can significantly reduce false negatives and improve identity continuity. OC-SORT [16] and its follow-up variants revisit motion modeling and observation-centric updates to better handle non-linear motion and imperfect detections. Other works [17,18,19] further incorporate adaptive measurement models, confidence-aware matching, or stronger appearance cues to improve tracking stability in crowded or dynamic scenes. Overall, 2D MOT has developed into a mature toolbox that provides temporally consistent object identities, reliable frame-to-frame associations, and efficient online inference.

From the perspective of 3D reconstruction, these properties are particularly attractive because MOT offers a lightweight way to aggregate object observations across multiple frames without requiring per-frame 2D segmentation masks. Instead of treating each image independently, MOT can provide long-range temporal correspondences for each target, allowing the system to collect object-specific image evidence over time. Such temporal consistency is valuable when the goal is not only to localize objects in 2D, but also to recover their corresponding 3D geometry from multiple viewpoints.

A small but emerging line of work has begun to explore tracking cues in 3D perception and point-cloud-related tasks, for example, by performing object tracking directly in point cloud sequences [20] or by using temporal association [21] to stabilize geometric estimation. However, most of these methods focus on 3D MOT in LiDAR/point cloud domains or on object trajectory estimation, rather than using 2D MOT as an intermediate mechanism for object-level point cloud extraction from multi-view image observations. In contrast, our work leverages 2D MOT as a lightweight temporal association module to gather target-consistent observations across views, and then uses these tracked object observations to guide point cloud extraction at the object level. This design avoids the heavy dependency on per-frame segmentation while naturally coupling temporal identity consistency with multi-view geometric reconstruction.

## 3. Methods

### 3.1. Overview

We propose a multi-object point cloud extraction pipeline that combines 2D MOT with monocular depth-based multi-view 3D reconstruction. The key idea is to avoid expensive per-object 3D segmentation by first exploiting efficient 2D MOT to obtain cross-view-consistent object identities in multiple views, and then lifting the tracked objects into 3D space through multi-view geometric association and point cloud refinement.

The observations are obtained from multiple calibrated camera positions. Our pipeline consists of four main stages:

1. 2D object detection and tracking: We use an MOT tracker to obtain object bounding boxes and track identities in each view.
2. Point cloud generation from monocular depth: For each image, a dense depth map is predicted, which is then back-projected into a camera-space point cloud and transformed into the global coordinate system.
3. Multi-view object association and coarse 3D localization: For each tracked object, the center of its 2D bounding box is converted into a 3D ray in each visible camera. Multiple rays corresponding to the same object are matched across views to estimate a coarse 3D object center. Based on this center, a dynamic spherical region is constructed to extract a coarse object-specific point subset.
4. 3D density-based denoising and refinement: Since the spherical proposal inevitably includes background points and outliers, we further perform 3D density estimation to suppress sparse noisy points and preserve the dense object structure, yielding the final extracted object point cloud.

**Figure 2:**
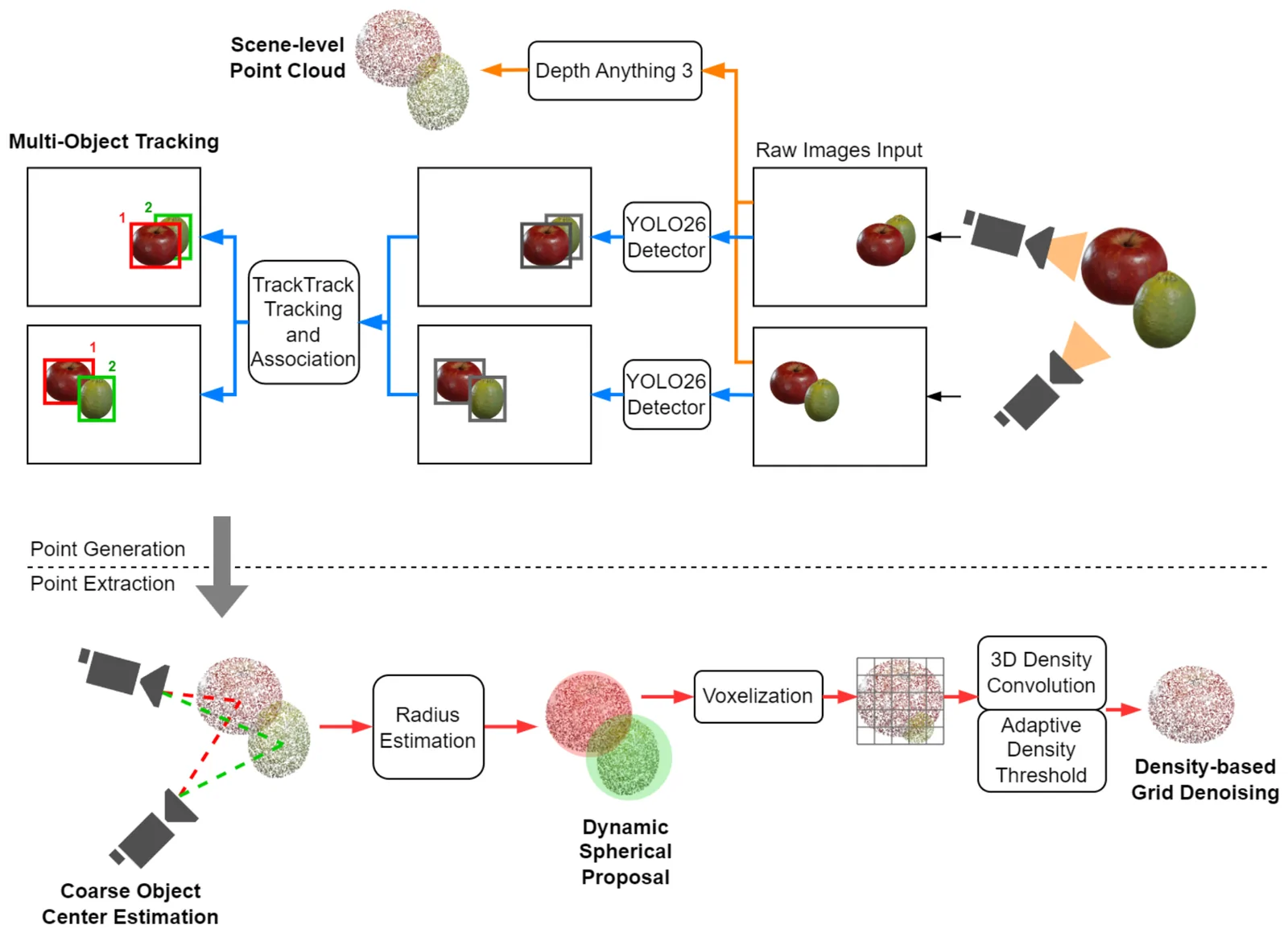
Overview of the proposed pipeline. Multi-view RGB images are processed by 2D MOT and monocular depth estimation to reconstruct a scene-level point cloud. Multi-view geometric association estimates coarse object locations, followed by density-based refinement to obtain object-specific point clouds.

Compared with direct 3D instance segmentation, the proposed pipeline leverages the efficiency of 2D MOT while still producing object-level 3D point clouds from multi-view observations. The proposed multi-object point cloud extraction pipeline is shown in Figure 2.

### 3.2. 2D Multi-Object Tracking

Let $I_{n}$ denote the *n*-th input image. We first perform 2D object detection which outputs a set of object bounding boxes:

(1) $$B_{n}={\left\{b_{n,m}\right\}}_{m=1}^{M_{n}},\;b_{n,m}=\left(x_{n,m}^{\min},y_{n,m}^{\min},x_{n,m}^{\max},y_{n,m}^{\max},c_{n,m},s_{n,m}\right),$$

where $M_{n}$ is the number of detected objects in image $I_{n}$, and each bounding box contains its spatial coordinates, category label $c_{n,m}$, and confidence score $s_{n,m}$.

To maintain object consistency across views or frames, we employ a tracking method to associate detections into trajectories. For the *m*-th tracked object, the set of observations across the views in which it is visible is written as

(2) $$T={\left\{T_{m}\right\}}_{m=1}^{M},\;T_{m}=\left\{\left(n,b_{n,m}\right)|n\in V_{m}\right\}.$$

where $V_{m}$ denotes the set of views in which object *m* is visible.

It should be noted that the proposed extraction strategy is modular and is not restricted to a specific MOT algorithm. In this work, the MOT module is only required to provide object bounding boxes and persistent track identities across views or frames. Therefore, different tracking methods can be integrated into the proposed pipeline as long as they provide consistent object identities for multi-view association. The tracking stage plays two important roles in our pipeline. First, it provides object-level grouping in the image domain, which avoids the need for dense 3D instance segmentation over the entire scene. Second, it ensures that 2D observations from different views belonging to the same physical object can be aggregated for subsequent 3D localization.

For each tracked box $b_{n,m}$, we define its 2D center as

(3) $$u_{n,m}=\left[\begin{array}{c} u_{n,m}\\ v_{n,m}\\ 1 \end{array}\right]=\left[\begin{array}{c} \left(x_{n,m}^{\min}+x_{n,m}^{\max}\right)/2\\ \left(y_{n,m}^{\min}+y_{n,m}^{\max}\right)/2\\ 1 \end{array}\right],$$

these center points are later used to generate object center rays for multi-view matching.

### 3.3. Monocular Depth-Based Point Cloud Generation

For completeness and reproducibility, this subsection describes how the common scene-level point cloud input is prepared in our experiments. The proposed object-level extraction method itself is independent of the specific point cloud generation technique.

For each input image $I_{n}$, we use a monocular depth prediction method to predict a dense depth map

(4) $$D_{n}=f_{\mathrm{depth}}\left(I_{n}\right),$$

where $D_{n}\left(u,v\right)$ denotes the estimated depth value at pixel (u,v). In our experiments, $D_{n}\left(u,v\right)$ is prepared using Depth Anything 3. This scene-point-cloud construction step is used only for experimental input preparation and is not a contribution of the proposed extraction method.

Using the camera intrinsic matrix

(5) $$K_{n}=\left[\begin{array}{ccc} f_{x} & 0 & c_{x}\\ 0 & f_{y} & c_{y}\\ 0 & 0 & 1 \end{array}\right],$$

each valid pixel (u,v) can be back-projected to a 3D point in the camera coordinate system:

(6) $$p_{n}^{c}\left(u,v\right)=D_{n}\left(u,v\right)\;K_{n}^{-1}\left[\begin{array}{c} u\\ v\\ 1 \end{array}\right],$$

let $p_{n}^{c}={\left[x_{n}^{c},y_{n}^{c},z_{n}^{c}\right]}^{T}$ be the camera-space coordinate. Given the camera pose $R_{n},t_{n}$, the point is transformed into the world coordinate system by

(7) $$p_{n}^{w}=R_{n}^{⊤}\left(p_{n}^{c}-t_{n}\right),$$

or equivalently, if the *n*-th camera-to-world transform $T_{n}^{cw}$ is directly available,

(8) $${\tilde{p}}_{n}^{w}=T_{n}^{cw}{\tilde{p}}_{n}^{c},$$

where $\tilde{p}$ denotes homogeneous coordinates.

By back-projecting all valid pixels from all views, we obtain a fused scene point cloud

(9) $$\Omega_{n}^{valid}=\left\{\left(u,v\right)|D_{n}\left(u,v\right)>0\right\}$$

(10) $$P=\overset{N}{\underset{n=1}{⋃}}P_{n},\;P_{n}=\left\{p_{n}^{w}\left(u,v\right)|\left(u,v\right)\in \Omega_{n}^{valid}\right\},$$

where $\Omega_{n}^{valid}$ is the set of valid depth pixels in image $I_{n}$.

### 3.4. Multi-View Object Association

After obtaining tracked 2D boxes and a scene-level point cloud, the next step is to associate the same tracked object across views and estimate its coarse 3D location.

#### 3.4.1. Object Center Ray Construction

For an object *m* observed in view *n*, we use the center of its bounding box $u_{n,m}$ as the image observation. This center is back-projected into a 3D ray originating from the camera center.

Let $C_{n}$ denote the *n*-th camera center in the world coordinate system. The ray direction corresponding to $u_{n,m}$ is computed as

(11) $$d_{n,m}=\frac{R_{n}^{⊤}K_{n}^{-1}u_{n,m}}{{∥R_{n}^{⊤}K_{n}^{-1}u_{n,m}∥}_{2}}.$$

Thus, the object center ray from camera *n* is defined as

(12) $$L_{n,m}\left(\lambda \right)=C_{n}+\lambda d_{n,m};\;\lambda >0.$$

Intuitively, each ray indicates the possible 3D positions of the tracked coarse object location consistent with its 2D box center in that view.

#### 3.4.2. Multi-View Ray Matching and Coarse Center Estimation

For each target object *m* observed in view *n*, since the rays from different views generally do not intersect exactly due to detection noise and geometric uncertainty, we estimate the coarse 3D center $c_{m}$ by minimizing the sum of squared distances from a 3D point to all rays:

(13) $$c_{m}=\arg\min_{x}\sum_{n\in V_{m}}{∥\left(I-d_{n,m}d_{n,m}^{⊤}\right)\left(x-C_{n}\right)∥}_{2}^{2},$$

where $I\in R^{3\times 3}$ denotes the 3×3 identity matrix.

Let

(14) $$P_{n,m}=I-d_{n,m}d_{n,m}^{⊤},\;n\in V_{m},$$

Then the above optimization can be solved in closed form as

(15) $$\left(\sum_{n\in V_{m}}P_{n,m}\right)c_{m}=\sum_{n\in V_{m}}P_{n,m}C_{n}.$$

Defining

(16) $$a_{m}=\sum_{n\in V_{m}}P_{n,m},\;b_{m}=\sum_{n\in V_{m}}P_{n,m}C_{n},$$

the coarse center is obtained by

(17) $$c_{m}={\left(a_{m}+\lambda_{reg}I\right)}^{-1}b_{m}.$$

where $\lambda_{reg}=10^{-6}$ is a small numerical regularization coefficient and $I\in R^{3\times 3}$ denotes the 3×3 identity matrix. This regularization improves numerical stability when the viewing-ray configuration produces a singular or ill-conditioned coefficient matrix.

Only views $n\in V_{m}$ with a valid observation of object *m* and valid camera calibration are included. After triangulation, the estimated center is accepted only when more than 60% of the valid viewing rays satisfy the adaptive ray-consistency criterion; otherwise the estimate is marked invalid.

It should be emphasized that the centers of the 2D bounding boxes are not assumed to correspond to the same physical surface point across views. Instead, each ray provides a coarse object-level line-of-sight constraint. Since occlusion and visible object extent vary with viewpoint, multi-view observations are complementary: a partially occluded or truncated observation in one view can be compensated by other views in which a larger portion of the object is visible. Therefore, the localization does not rely on a single box center. Equation (13) combines all available rays in a least-squares sense rather than enforcing an exact intersection, thereby reducing sensitivity to view-specific box-center shifts.

### 3.5. Dynamic Spherical Proposal

Although the coarse object center $c_{m}$ provides an approximate object location, it is insufficient for directly extracting the corresponding object point cloud, since the reconstructed scene point cloud contains points belonging to multiple objects as well as background structures. Therefore, we construct a spherical proposal centered at $c_{m}$ to obtain a coarse candidate point set for each object.

#### 3.5.1. Dynamic Radius Estimation

For each target object *m*, an adaptive spherical proposal is constructed around the triangulated coarse object center $c_{m}$. The sphere radius is estimated according to both the projected object scale and the observation uncertainty across multiple views.

Given the triangulated coarse object center $c_{m}$, the distance between the object and the camera in the *n*-th view is defined as

(18) $$\rho_{n,m}={∥c_{m}-C_{n}∥}_{2},$$

where $C_{n}$ denotes the *n*-th camera center in the world coordinate system.

Given the 2D bounding box of object *m* in view *n*, its width $w_{n,m}$ and height $h_{n,m}$ are defined as

(19) $$w_{n,m}=x_{n,m}^{\max}-x_{n,m}^{\min},\;h_{n,m}=y_{n,m}^{\max}-y_{n,m}^{\min}.$$

Based on the pinhole camera model, the basic sphere radius is estimated as

(20) $$r_{n,m}^{\mathrm{base}}=\frac{1}{2}\sqrt{{\left(\frac{\rho_{n,m}w_{n,m}}{f_{n,x}}\right)}^{2}+{\left(\frac{\rho_{n,m}h_{n,m}}{f_{n,y}}\right)}^{2}},$$

where $f_{n,x}$ and $f_{n,y}$ denote the horizontal and vertical focal lengths of the intrinsic camera matrix for the *n*-th view, respectively.

The basic radius only reflects the projected object size and does not account for the increasing localization uncertainty of distant or poorly observed objects. To improve the robustness of the spherical proposal, we further introduce an adaptive inflation factor.

Specifically, the projected bounding-box scale in the *n*-th view is defined as

(21) $$s_{n,m}=\sqrt{w_{n,m}\;\cdot \;h_{n,m}}.$$

For each object, a reference projected scale is then computed from all valid observations using the median operator:

(22) $${\bar{s}}_{m}={\mathrm{median}}_{n}\left(s_{n,m}\right).$$

To model the influence of the camera–object distance, we define the distance factor as

(23) $$\phi_{n,m}^{dist}=\frac{\rho_{n,m}}{\rho_{n,m}+\tau }.$$

Here, τ is a length-scale parameter with the same physical unit as $\rho_{n,m}$ and the 3D point cloud coordinates. All coordinates used in the dynamic-radius computation are expressed in meters, and we set τ=5.0 m as the default value in all experiments. No object-wise or dataset-wise scale normalization, such as unit-sphere or unit-bounding-box normalization, is applied.

Similarly, a size factor is introduced to compensate for observations with relatively small projected bounding boxes:

(24) $$\phi_{n,m}^{size}={\left(\frac{{\bar{s}}_{m}}{s_{n,m}}\right)}^{\gamma }.$$

where γ is a saturation parameter that determines the sensitivity of the weighting to bounding box size variations.

The adaptive inflation factor is then computed as

(25) $$\eta_{n,m}=1+\beta \;\cdot \;\phi_{n,m}^{dist}\;\cdot \;\phi_{n,m}^{size}.$$

where β denotes the overall inflation coefficient controlling the strength of the dynamic radius adjustment.

The sphere radius for object *m* in the *n*-th view is therefore given by

(26) $$r_{n,m}=r_{n,m}^{\mathrm{base}}\;\cdot \;\eta_{n,m}.$$

Finally, we aggregate the per-view radius across all valid observations using a median operator to obtain the final sphere radius for object *m*:

(27) $$r_{m}={\mathrm{median}}_{n}\left(r_{n,m}\right).$$

In this way, the proposed sphere radius is jointly determined by the projected object scale, the camera–object distance, and the relative observation quality of each view, making it more suitable for coarse object-level point cloud extraction under multi-view tracking noise and scale variation.

#### 3.5.2. Spherical Point Proposal

Given the coarse center $c_{m}$ and dynamic radius $r_{m}$, we extract a coarse object point set by selecting all scene points within the sphere:

(28) $$Q_{m}=\left\{p\in P\;|\;{∥p-c_{m}∥}_{2}\leq r_{m}\right\}.$$

This spherical proposal provides an efficient object-centric search region. It is computationally cheap and does not depend on accurate object masks or complete frustum intersection. However, because the sphere is intentionally enlarged to preserve object completeness, $Q_{m}$ may still contain irrelevant background points, especially when nearby objects or scene surfaces lie close to the target. Therefore, an additional refinement stage is required.

The spherical proposal also provides tolerance to moderate errors in the estimated anchor. Its radius is derived from the projected object scale and camera–object distance over multiple observations and is intentionally inflated before median aggregation. Therefore, even when the anchor is slightly displaced because of asymmetry or partial occlusion, the target points can still remain inside the candidate region. The following density-based refinement then relies on the actual 3D point distribution, rather than the precise anchor location, to preserve compact object structures and suppress sparse background points.

### 3.6. Density-Based Grid Denoising

Although the dynamic spherical proposal significantly narrows the spatial search range for each object, the extracted candidate point set may still contain background clutter and sparse outliers caused by tracking inaccuracies, coarse center estimation, and monocular depth noise. To further refine the object-level point cloud, we perform a density-based denoising step in a discretized 3D grid space.

For each object, the points contained within the spherical proposal form a candidate point set

(29) $$Q_{m}={\left\{p_{i}\right\}}_{i=1}^{N_{m}},$$

where $N_{m}$ denotes the number of candidate points.

The local object space is then discretized into a regular voxel grid of size $\left(G_{1},G_{2},G_{3}\right)$. Each point is assigned to a voxel according to its spatial coordinates, resulting in a voxel count grid

(30) $${Vox}_{m}\in N^{G_{1}\times G_{2}\times G_{3}},$$

where each element of ${Vox}_{m}$ records the number of points contained in the corresponding voxel.

To estimate the local 3D density distribution, we convolve the voxel count grid with a fixed kernel:

(31) $$E_{m}={Vox}_{m}*K,$$

where * denotes 3D convolution and kernel $K\in R^{3\times 3\times 3}$ is defined as

(32) $$K_{1}=\left[\begin{array}{ccc} 1 & 2 & 1\\ 2 & 4 & 2\\ 1 & 2 & 1 \end{array}\right],\;K_{2}=\left[\begin{array}{ccc} 2 & 4 & 2\\ 4 & 8 & 4\\ 2 & 4 & 2 \end{array}\right],\;K_{3}=\left[\begin{array}{ccc} 1 & 2 & 1\\ 2 & 4 & 2\\ 1 & 2 & 1 \end{array}\right].$$

This kernel assigns larger weights to the center voxel and its nearby neighbors, so that compact object regions with consistently high point counts receive stronger responses, while isolated voxels or sparse background structures remain weakly activated.

Let

(33) $$A_{m}=\left\{E_{m}\left(u,v,w\right)|E_{m}\left(u,v,w\right)>0,\;1\leq u\leq G_{1},\;1\leq v\leq G_{2},\;1\leq w\leq G_{3}\right\},$$

denote the set of non-zero density responses for object *m*. We first compute the median density

(34) $$\mu_{m}=\mathrm{median}\left(A_{m}\right),$$

and the median absolute deviation (MAD)

(35) $${\mathrm{MAD}}_{m}=\mathrm{median}\left(\left|a-\mu_{m}\right|;a\in A_{m}\right).$$

Based on these statistics, we define a normalized spread factor

(36) $$s_{m}=\frac{{\mathrm{MAD}}_{m}}{\mu_{m}}.$$

To adapt the filtering strength to different density distributions, we determine the density threshold according to the local density statistics of each object. Specifically, the quantile level is computed as

(37) $$q_{m}=clip\left(0.95-s_{m},0,1\right),$$

a larger spread factor $s_{m}$ indicates a more dispersed density distribution, resulting in a lower quantile to avoid over-pruning fragmented yet valid object structures. Conversely, a smaller spread factor leads to a higher quantile, preserving only the densest object regions.

The density threshold is then determined as the $q_{m}$-quantile of the non-zero density responses:

(38) $$T_{m}=Q_{q_{m}}\left(A_{m}\right),$$

where $Q_{q_{m}}\left(\;\cdot \;\right)$ denotes the quantile operator.

Based on the estimated threshold, voxels whose density responses exceed the threshold are retained:

(39) $$M_{m}\left(u,v,w\right)=1\;\left(E_{m}\left(u,v,w\right)\geq T_{m}\right),$$

where $M_{m}$ denotes the binary voxel mask.

Finally, the remaining points constitute the filtered object point cloud ${\tilde{P}}_{m}$.

The above procedure preserves the compact high-density structure of each object while effectively suppressing scattered outliers and background points. Since the density threshold is adaptively determined from the object-specific density distribution, the proposed denoising strategy is robust to scale variation and point sparsity across different objects and scenes.

## 4. Experiments

### 4.1. Setup

We evaluate the proposed MOT-based object point cloud extraction pipeline on both single-object and multi-object scenarios. To evaluate the feasibility of the proposed method under complementary acquisition and scene conditions, we conduct experiments on representative samples from DTU [22], MVImgNet [23], and ScanNet++ [24].

For single-object extraction, we use DTU and MVImgNet. These datasets provide multi-view object observations and are suitable for evaluating the geometric quality of the extracted object point clouds under controlled object-centric settings. In particular, DTU is used as the main benchmark for quantitative evaluation due to its accurate multi-view acquisition and high-quality geometry, while MVImgNet is used to further verify the cross-dataset applicability of the proposed method in real-world environments.

For multi-object extraction, we use ScanNet++, which contains realistic indoor scenes with multiple objects, clutter, occlusion, and complex backgrounds. This setting is used to evaluate whether the proposed pipeline can simultaneously extract point clouds for multiple tracked objects from a shared scene reconstruction, which is difficult for conventional per-frame segmentation-based pipelines to achieve efficiently.

**Figure 3:**
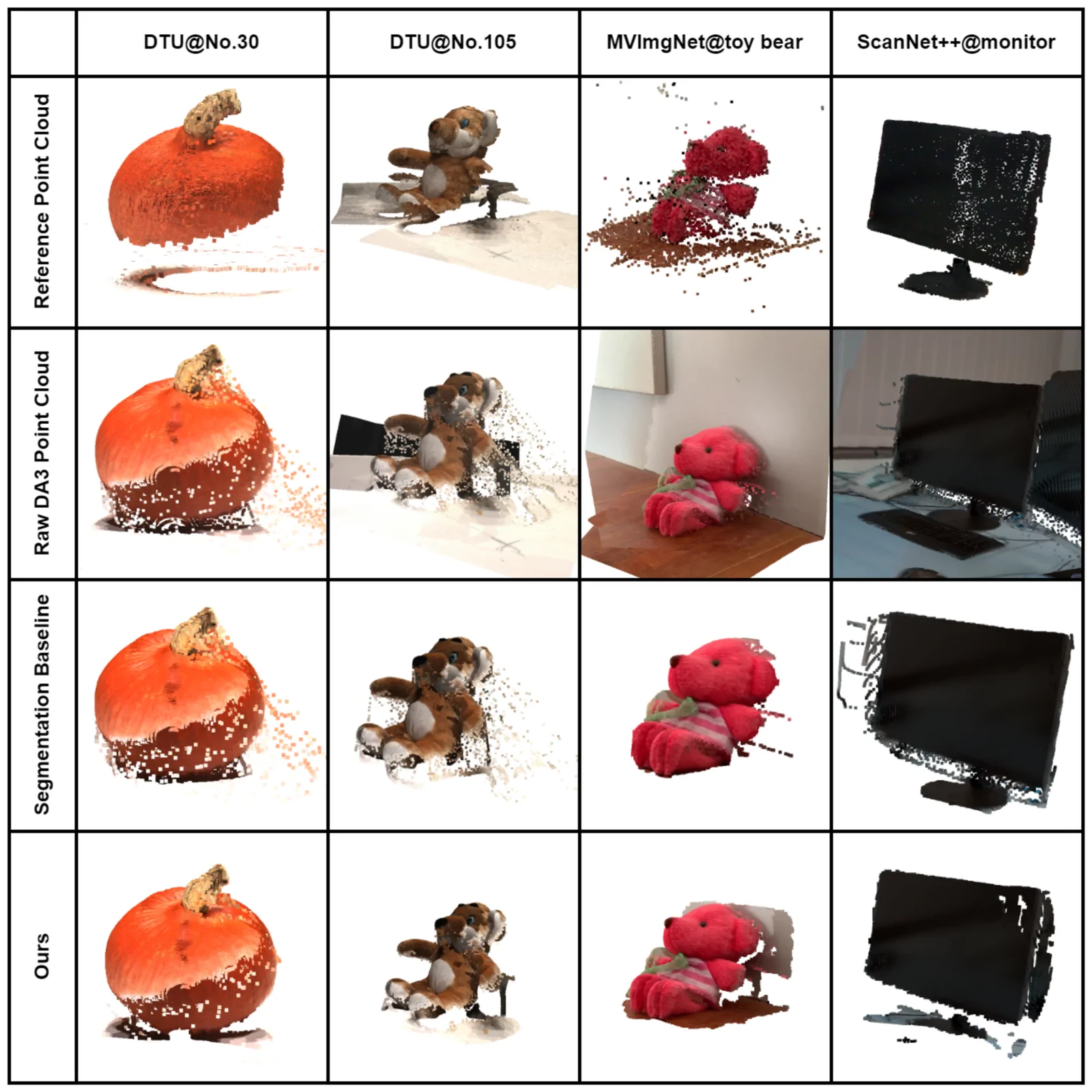
Qualitative result comparison on DTU Sequence 30, DTU Sequence 105, the toy bear category of MVImgNet, and the monitor category of ScanNet++ with dataset-provided reference point clouds. One target object is displayed for each dataset to maintain a consistent visualization layout.

For all datasets, the reference point clouds used as ground truth (GT) in the quantitative evaluation and Figure 3 are directly obtained from the corresponding dataset releases [22,23,24]. For each sample, the reference point cloud corresponding to the evaluated target object is used, without any additional reconstruction of the GT geometry.

All evaluations were conducted using a single NVIDIA GeForce RTX 3090 GPU and an Intel Core i9-12900K CPU.

#### 4.1.1. Reconstruction and Tracking Pipeline

We first estimate dense depth maps using Depth Anything 3 [25] to obtain a scene-level point cloud. Then, 2D object trajectories across views are obtained using the MOT module composed of YOLO26 [26] for object detection and TrackTrack [19] for identity association. Based on the tracked object boxes, we establish cross-view object correspondence and extract object-specific 3D points by the proposed multi-view ray-based matching and spatial filtering strategy described in Section 3.

The evaluated datasets do not provide hardware-synchronized multi-camera observations. Therefore, we manually sample three non-contiguous images as input to simulate different views for each evaluation. Each image input is sampled at an interval of at least 10 frames. Only the selected views are processed; intermediate frames are not used. The target objects and surrounding scene geometry are assumed to remain static or approximately static across the selected views. The view order is used for persistent identity association rather than for modeling physical object motion. We do not rely on continuous video streams; instead, our method is designed for sparse multi-view inputs. Therefore, using non-contiguous viewpoints in the experiments is more consistent with the intended problem setting. Different camera settings are adopted for different datasets. On DTU, calibrated camera intrinsics and extrinsics are used to evaluate performance under ideal conditions. On MVImgNet, camera parameters estimated by Depth Anything 3 are employed to mimic real-world deployment. On ScanNet++, calibrated camera parameters are used to simulate robotic perception scenarios. Only the three selected observations are processed by the tracking and object extraction pipeline; intermediate frames are not used.

As YOLO26 is trained on the COCO categories [27], the available evaluation samples are constrained by the overlap between COCO object categories and the objects contained in 3D reconstruction datasets. In addition, quantitative evaluation requires multi-view observations, usable camera geometry, and an object-level reference point cloud. Existing datasets rarely satisfy all of these requirements simultaneously. Specifically, we use Scan 30 and Scan 105 from the DTU dataset, sequences from the “toy bear” category in MVImgNet, and scenes containing “monitor” objects from ScanNet++. The downstream ray aggregation, spherical proposal, and density-refinement stages do not use category-specific geometric parameters once bounding boxes and Track IDs are available. No category-specific extraction parameters are tuned for the evaluated samples. Therefore, we select representative object categories with relatively complex geometry that are also convenient for the detector to handle, in order to facilitate stable initialization while still providing a challenging setting for multi-view reconstruction and tracking consistency evaluation.

**Figure 4:**
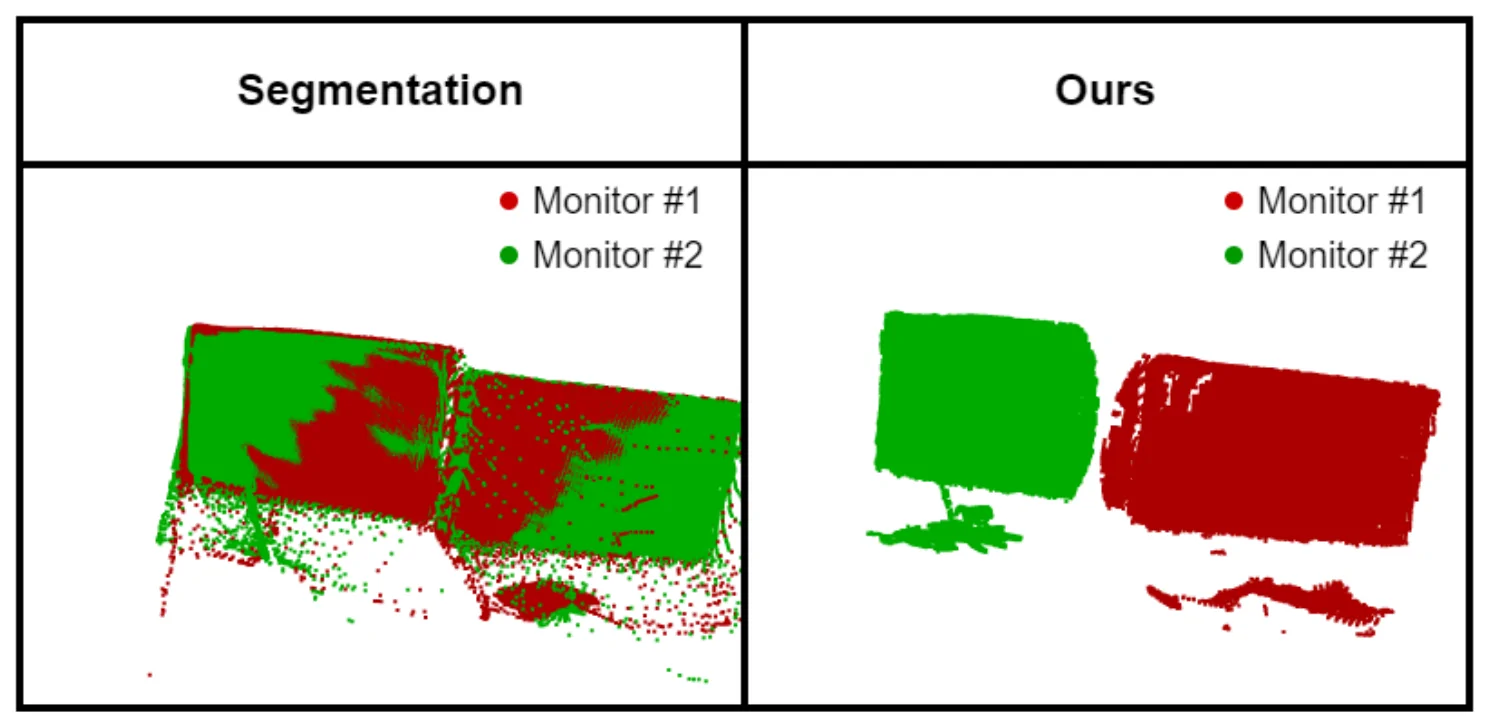
Illustration of point cloud extraction for the monitor category on ScanNet++, comparing a segmentation-only method with our approach for simultaneous multi-view, multi-object point cloud extraction.

For the cross-dataset qualitative comparison in Figure 3, only one representative ScanNet++ monitor instance is displayed to maintain a consistent single-object visualization layout across datasets. This visualization choice is independent of the dedicated multi-object evaluation in Section 4.5, where Figure 4 simultaneously presents two monitor instances with distinct Track IDs.

Unless otherwise specified, all experiments use the same reconstruction and tracking settings across datasets. For a fair comparison, the same camera poses and the same depth estimation pipeline are used for all compared object extraction methods.

#### 4.1.2. Evaluation Metrics

To evaluate the quality of extracted object point clouds, we adopt standard geometric reconstruction metrics, including Chamfer Distance (CD), Precision, Recall, and F-score. CD measures the bidirectional nearest-neighbor distance between the predicted and ground-truth point clouds. Precision and Recall quantify the fractions of predicted points and ground-truth points, respectively, whose nearest-neighbor distances are below a predefined threshold. F-score is computed as the harmonic mean of Precision and Recall. For the DTU benchmark, we additionally report the Overall score following the official evaluation protocol. For MVImgNet and ScanNet++, F-score@1% denotes the F-score computed with a distance threshold equal to 1% of the diagonal length of the ground-truth bounding box. Overall is defined as the average of the Accuracy and Completeness distances, providing a comprehensive measure of reconstruction quality. Lower Overall values indicate better reconstruction performance.

For efficiency analysis, we additionally report runtime results as the processing time without scene-level point cloud generation. The reported runtime does not include monocular depth prediction or point cloud reconstruction, and only accounts for the object-level point extraction stage. This stage includes the method-specific object cue generation, namely dense instance segmentation for the segmentation baseline and 2D detection/MOT association for the proposed method. These results are used to compare the proposed MOT-based pipeline with segmentation-based baselines in terms of computational efficiency.

A separate MOT benchmark is not reported because the evaluated datasets do not provide identity annotations for the adopted sparse-view protocol. Moreover, DTU and MVImgNet contain only one evaluated target instance per sample, making conventional Track-ID metrics uninformative. TrackTrack is used as a fixed upstream module, and its association errors are included in the reported end-to-end object-point-cloud extraction results.

The reference point clouds used for quantitative evaluation and visualization are obtained from the corresponding official dataset releases. For DTU, the predicted point clouds are evaluated following the official DTU evaluation protocol, including unit conversion, valid observation-region filtering, and ground-plane filtering. For MVImgNet, the reference geometry is obtained from the dataset-provided COLMAP reconstruction. For quantitative evaluation only, a single global similarity transformation is estimated by applying Umeyama alignment to the corresponding camera centers derived from the DA3-predicted camera poses and the dataset-provided COLMAP camera poses of the three selected views. The estimated rotation, translation, and scale are then applied uniformly to the entire DA3-derived scene point cloud before computing CD, Precision, Recall, and F-score. No point-level correspondences, ICP, object-specific registration, or method-specific alignment are used to estimate this transformation. For ScanNet++, the corresponding dataset-provided reference point clouds are used directly, and 3D instance annotations are used for multi-object evaluation. No additional scale alignment, rigid registration, ICP, or point resampling is applied. The reference point clouds are used only for evaluation and visualization and are not involved in object detection, tracking, coarse localization, spherical proposal generation, or density-based refinement.

#### 4.1.3. Implementation Details

We implement the method as a multi-component pipeline integrating detection, tracking, depth estimation, and 3D reconstruction. For 2D object detection, we adopt YOLO26X, where the confidence threshold is set to 0.6 and the NMS IoU threshold is 0.7.

For multi-object tracking, we use TrackTrack, and extract appearance features using the FastReID SBS (S50) backbone.

For monocular depth estimation, we employ Depth Anything 3, specifically the DA3-Nested-Giant-Large configuration. All input images are resized to 504×378 before depth inference. For YOLO26X, images are additionally resized to 640×480 for detection.

In the Dynamic Radius Estimation stage, we set the hyperparameters as β=1.0, τ=5.0 m, and γ=0.5. The voxel grid used for density-based refinement is fixed at a resolution of (64×64×64).

All experiments are conducted under the following environment: GPU driver version 595.95, CUDA toolkit 12.8.61, and PyTorch 2.10.0.

### 4.2. Baselines and Compared Settings

We compare the proposed MOT-based object extraction pipeline with segmentation-based alternatives to analyze the trade-off between object-level extraction quality, efficiency, and multi-object capability.

We consider a “Scene-level Baseline”, which directly reconstructs a single unified point cloud of the entire scene without any object-level processing or post-processing object-level extraction. This baseline serves as a reference to evaluate how much object-level decomposition benefits individual object reconstruction, as it does not perform segmentation, tracking, or per-object point cloud separation.

For the segmentation comparison, we intentionally adopt the segmentation version of YOLO26X as a closely matched baseline. The proposed pipeline uses the YOLO26X object-detection model followed by TrackTrack, whereas the baseline replaces the detection-and-tracking cue with dense instance masks predicted by YOLO26X-seg. Because the two alternatives belong to the same model family and use comparable model scales, training categories, input resolutions, and implementation environments, this setting minimizes architectural and implementation-related confounding factors. Both methods further share the same input images, camera parameters, DA3-generated scene point clouds, and evaluation procedure. Therefore, the comparison primarily reflects the difference between MOT-based bounding-box/identity cues and segmentation-based pixel-mask cues, rather than differences between unrelated network architectures. In the segmentation comparison experiments, this baseline is referred to as the “Segmentation Baseline”. The predicted masks are projected into 3D to extract the target object point cloud. Owing to its more accurate object boundaries, this baseline is expected to produce cleaner object point clouds in single-object scenarios.

#### Ablation Settings

To analyze how the proposed pipeline progressively converts a fixed scene-level point cloud into an object-specific point cloud, we evaluate three cumulative settings on DTU.

DA3 Raw Scene Cloud: The DA3-generated scene-level point cloud is directly evaluated without object-level association, spatial proposal, or density refinement.

MOT-Guided Object Extraction: Persistent Track IDs are used to group detections belonging to the same object across views. The grouped observations are then used for multi-view ray aggregation, coarse 3D object localization, and dynamic spherical point proposal. Density-based denoising is disabled in this setting.

MOT + Denoising: This is our full method. Density-based grid denoising is further applied to the MOT-guided candidate point cloud.

In the proposed pipeline, MOT association and geometric object proposal are functionally coupled because the Track IDs determine which multi-view observations and viewing rays are aggregated for each object. Therefore, the comparison between the Raw Scene Input and MOT-Guided Object Extraction evaluates the overall contribution of introducing the MOT-assisted object-level extraction stage, rather than the isolated performance of the tracking algorithm. The comparison between MOT-Guided Object Extraction and the MOT + Denoising full method isolates the additional contribution of density-based refinement.

### 4.3. Single-Object Point Cloud Extraction

We first evaluate the proposed method on single-object extraction using DTU and MVImgNet.

#### 4.3.1. Results on DTU

Table 1 shows the effectiveness of object-level extraction from a fixed DA3-generated scene point cloud on DTU. The raw scene input contains the target object together with surrounding floors, walls, neighboring objects, and other background points. Therefore, its comparison with the object-specific GT is intended to quantify target isolation and background suppression. The proposed method successfully extracts object-centric point clouds from multi-view observations. Specifically, on Sequence 30, the proposed method reduces the CD from 90.97 mm to 12.52 mm, while the F-score increases from 0.4601 to 0.9289. Precision is substantially improved from 0.2998 to 0.8776, with Recall remaining consistently high (0.9882 vs. 0.9866). The Overall metric is also reduced from 2.055 to 1.285. Similar improvements are observed on Sequence 105, where CD decreases from 15.31 mm to 6.92 mm, F-score increases from 0.8468 to 0.9057, and Precision improves from 0.7629 to 0.8666 while maintaining comparable Recall (0.9514 vs. 0.9485). The Overall metric is further reduced from 1.848 to 1.600. These results demonstrate that stable MOT-based object association effectively suppresses mismatched background points and improves the spatial consistency of the extracted object point clouds.

#### 4.3.2. Results on MVImgNet

We further validate the proposed method on MVImgNet, and the results are shown in Table 2. Similar trends are observed across both datasets. On the toy bear category, the proposed method reduces the Chamfer Distance from 1.23 m to 0.46 m. Meanwhile, the F-score increases from 0.6457 to 0.9323, and Precision improves remarkably from 0.4777 to 0.9337. Although Recall decreases slightly from 0.9959 to 0.9310, it remains high while producing a much better balance between Precision and Recall, resulting in a significantly higher object-level extraction quality. These results show that the same fixed pipeline and hyperparameter configuration remain applicable to the evaluated real-world MVImgNet sample. Together with the controlled DTU evaluation, this result provides cross-dataset evidence under the tested acquisition conditions, rather than exhaustive category-level generalization.

Qualitative results in Figure 3 show that the extracted point clouds preserve the main object geometry and remove a large portion of surrounding clutter.

### 4.4. Ablation Study

We conduct ablation studies on DTU to analyze the contribution of each component in the proposed pipeline.

Table 3 compares the raw DA3-generated scene point cloud with the object-specific point subset obtained through MOT-guided cross-view association and the dynamic spherical proposal. This comparison evaluates the overall effect of introducing object-level extraction, rather than the isolated contribution of the tracking algorithm alone.

#### 4.4.1. Effect of MOT-Guided Object-Level Extraction

We first evaluate the effect of introducing the integrated MOT-assisted object-level extraction stage. As shown in Table 3, introducing the MOT-based cross-view association substantially improves the extraction quality on both sequences. On Sequence 30, the Chamfer Distance decreases from 90.97 mm to 27.22 mm, while the F-score increases from 0.4601 to 0.8738. Precision is significantly improved from 0.2998 to 0.7831, with Recall remaining unchanged at 0.9882. The Overall metric is also reduced from 2.055 to 1.387. Similar improvements are observed on Sequence 105, where the Chamfer Distance decreases from 15.31 mm to 7.31 mm, the F-score increases from 0.8468 to 0.8999, and Precision improves from 0.7629 to 0.8539, while Recall remains nearly identical (0.9514 vs. 0.9513). The Overall metric is further reduced from 1.848 to 1.615. These results show that the integrated MOT-guided grouping and geometric proposal stage substantially suppress non-target scene points relative to the no-extraction reference. Because Track-ID grouping and geometric proposal are functionally coupled, this comparison does not isolate the contribution of the tracker alone. Without object-level association and proposal generation, the raw scene input retains substantial background and neighboring-scene geometry and therefore cannot directly serve as an object-specific point cloud.

#### 4.4.2. Effect of Density-Based Denoising

We then evaluate the density-based denoising module by removing it from the pipeline. Without this step, the extracted point clouds contain significantly more outliers, especially around object boundaries and in cluttered regions. Quantitatively, on Sequence 30, the density refinement further reduces the Chamfer Distance from 27.22 mm to 12.52 mm, increases the F-score from 0.8738 to 0.9289, and improves Precision from 0.7831 to 0.8776, while Recall remains nearly unchanged (0.9882 vs. 0.9866). The Overall metric is also reduced from 1.387 to 1.285. On Sequence 105, the Chamfer Distance is further reduced from 7.31 mm to 6.92 mm, the F-score increases from 0.8999 to 0.9057, Precision improves from 0.8539 to 0.8666, and Recall changes only slightly from 0.9513 to 0.9485. Meanwhile, the Overall metric decreases from 1.615 to 1.600. These results confirm that the density refinement effectively suppresses noisy points retained after coarse geometric filtering, leading to cleaner and more geometrically consistent object point clouds.

Overall, the ablation study demonstrates that the final performance gain does not come from a single component, but from the combination of MOT-based association, adaptive 3D candidate selection, and density-aware refinement.

#### 4.4.3. Sensitivity Analysis of Dynamic-Radius Parameters

To evaluate the robustness of the dynamic spherical proposal to its hyperparameter settings, we conduct a one-factor-at-a-time sensitivity analysis on DTU Sequences 30 and 105. Specifically, the overall inflation coefficient is varied as $\beta \in \left\{0.5,1.0,1.5\right\}$, the distance saturation parameter as $\tau \in \left\{2.5,5.0,10.0\right\}$ m, and the projected-scale exponent as $\gamma \in \left\{0.25,0.5,1.0\right\}$, while the remaining parameters, depth maps, camera poses, detection boxes, Track IDs, and scene-level point clouds are kept fixed. All results are shown in Table 4.

On Sequence 30, varying β results in Chamfer Distance of 11.54 to 12.91 mm and F-scores of 0.9256 to 0.9353, whereas varying τ results in Chamfer Distance of 11.65 to 13.04 mm and F-scores of 0.9249 to 0.9346. On Sequence 105, the corresponding ranges are 6.49 to 7.14 mm and 0.9025 to 0.9094 for β, and 6.52 to 7.40 mm and 0.9011 to 0.9089 for τ. For each parameter sweep, the variation in Recall remains below 0.0009, indicating that target completeness is largely preserved and that β and τ mainly affect the trade-off between proposal size, background inclusion, and Precision. In comparison, the method is substantially less sensitive to γ: varying γ from 0.25 to 1.0 changes the Chamfer Distance by at most 0.0003 mm on Sequence 30 and 0.041 mm on Sequence 105, while the maximum F-score variation is below 0.0004. This limited sensitivity is consistent with the relative-scale factor and the median aggregation of the per-view radii, which suppress the influence of atypical bounding-box scales. Although a smaller β or a larger τ yields slightly better Chamfer Distance and Precision on these two sequences, the default configuration (β,τ,γ)=(1.0,5.0,0.5) is retained as a central setting and is applied uniformly without sequence-specific tuning. Overall, the results demonstrate that the proposed extraction method remains stable over the evaluated parameter ranges.

### 4.5. Multi-Object Extraction in ScanNet++

We further evaluate the proposed method in a more challenging multi-object setting on ScanNet++. Unlike object-centric datasets that mainly contain a single foreground object, ScanNet++ provides cluttered indoor scenes with multiple objects, occlusion, and shared scene geometry. This setting is used to evaluate whether the proposed pipeline can simultaneously maintain separate Track IDs and extract multiple object-specific point clouds from a shared scene-level point cloud.

#### Multi-Object Extraction on ScanNet++

Figure 4 presents representative examples on ScanNet++. Our method can extract object-specific point clouds for all tracked targets within a unified pipeline. Even in cluttered indoor scenes, the proposed method successfully separates multiple objects and extracts individual object point clouds while preserving object identities across views. For a fair comparison, the segmentation-based results are manually associated across multiple views and objects to establish consistent object correspondences.

Table 5 further compares our method with a segmentation-based baseline. The segmentation-based method achieves an F-score of 0.5315 and a Precision of 0.5698, which are slightly higher than those of our method (0.5188 and 0.5626, respectively), benefiting from more accurate object boundary estimation. Meanwhile, our method achieves a slightly lower Chamfer Distance of 0.027 m compared with 0.028 m for the segmentation-based baseline, while maintaining a comparable Recall (0.4812 vs. 0.4981).

Overall, the object-level extraction quality of the proposed method remains competitive with the segmentation-based approach. More importantly, our method naturally supports simultaneous extraction of multiple objects, whereas the segmentation-based baseline produces accurate per-frame masks but requires an additional cross-view association module. Cross-view identity association is non-trivial, and existing solutions typically introduce additional complexity and are less straightforward to integrate in a unified manner. Unlike segmentation, which predicts each object independently, MOT explicitly maintains persistent object identities across the image sequence, allowing all tracked objects to be extracted consistently from a single reconstructed scene.

### 4.6. Runtime Analysis

The runtime study is designed as a controlled comparison rather than an exhaustive benchmark across heterogeneous segmentation systems. The proposed method uses the object-detection version of YOLO26X, while the baseline uses its corresponding segmentation version. This closely matched configuration controls for model family, model scale, training taxonomy, input setting, implementation environment, and hardware, allowing the comparison to focus on the difference between detection/MOT-based object cues and dense segmentation masks.

We compare the runtime of the proposed MOT-based extraction and the segmentation-based baseline in both single-object and multi-object settings. The results are summarized in Table 6.

In the single-object setting, the segmentation-based method can achieve slightly better extraction quality due to its pixel-level masks, but its computational cost is higher because dense segmentation must be performed for each frame. On DTU Sequence 30, the segmentation-based baseline achieves a CD of 5.41 mm, an F-score of 0.9831, and an Overall score of 1.190, while requiring 0.3659 s for each group of three input frames. In comparison, the proposed method achieves a CD of 12.52 mm, an F-score of 0.9289, and an Overall score of 1.285, while reducing the runtime to 0.3365 s. Similar observations can be made on DTU Sequence 105, where the runtime decreases from 0.3708 s to 0.3405 s, while maintaining competitive object-level extraction quality (CD: 3.48 mm vs. 6.92 mm, F-score: 0.9539 vs. 0.9057, Overall: 1.474 vs. 1.600). On the MVImgNet toy bear category, both methods obtain the same CD of 0.46 m while the F-scores are respectively 0.9242 and 0.9323, and the proposed method reduces the runtime from 0.3907 s to 0.3569 s.

In the multi-object setting, the proposed method achieves a lower measured runtime in the evaluated example. On ScanNet++, the proposed method reduces the runtime from 0.3722 s to 0.3254 s while maintaining comparable object-level extraction quality (CD: 0.028 m vs. 0.027 m and F-score: 0.5315 vs. 0.5188). Since MOT directly provides persistent identities for multiple targets, the same tracking results can be reused to extract multiple object point clouds from a single reconstructed scene. Since the scene-level point cloud and MOT outputs are shared, multiple Track IDs can be processed within the same pipeline without repeating scene reconstruction or introducing a separate post hoc cross-view identity-association stage. In contrast, segmentation-based methods rely on dense per-frame mask prediction, per-instance mask projection, and an additional cross-view identity-association procedure for track-consistent multi-object extraction.

These results indicate an accuracy–efficiency trade-off under the evaluated settings. Although the segmentation baseline often provides more accurate object boundaries, the MOT-assisted pipeline achieves an approximately 8.0–12.6% lower measured object-extraction runtime in the tested cases and provides built-in cross-view Track-ID association. A segmentation-only pipeline would require an additional cross-view identity-association stage to provide the same track-consistent multi-object output.

## 5. Conclusions

In this paper, we presented an MOT-assisted pipeline for object-level point cloud extraction from multi-view observations. Instead of relying on computationally expensive per-frame instance segmentation, the proposed approach exploits temporally consistent 2D multi-object tracking to establish cross-view object correspondences and combines them with monocular depth-based scene reconstruction to recover object-specific point clouds. To improve extraction quality, we further introduced a multi-view geometric association strategy for coarse object localization, an adaptive spherical proposal for candidate point selection, and a density-based refinement module for suppressing background noise and outliers.

Experiments on DTU, MVImgNet, and ScanNet++ demonstrate the effectiveness of the proposed pipeline. On the DTU benchmark, the proposed method reduced the Chamfer Distance from 90.97 mm to 12.52 mm on Sequence 30 and achieved an F-score of 0.9289. The substantial increase in Precision, together with the nearly unchanged Recall, demonstrates that the method effectively suppresses non-target scene points while preserving most of the valid target geometry. On MVImgNet, the Chamfer Distance was reduced from 1.23 m to 0.46 m, with the F-score increasing from 0.6457 to 0.9323, providing evidence of applicability under the evaluated real-world setting. In the more challenging multi-object setting of ScanNet++, the proposed pipeline achieved extraction quality comparable to the evaluated segmentation baseline in the tested ScanNet++ example while naturally supporting simultaneous extraction of multiple objects within a unified pipeline. Moreover, the proposed method yielded modest but consistent reductions in the measured object-extraction runtime across the evaluated cases, achieving runtime reductions from 0.3659 s to 0.3365 s on DTU Sequence 30, from 0.3708 s to 0.3405 s on DTU Sequence 105, from 0.3907 s to 0.3569 s on MVImgNet and from 0.3722 s to 0.3254 s on ScanNet++, demonstrating a favorable trade-off between object-level extraction quality and computational efficiency.

The proposed method also has several limitations. Since the coarse 3D object center is estimated from rays generated by 2D bounding-box centers, localization errors may occur for non-symmetric, elongated, partially occluded, or inaccurately detected objects. The multi-view formulation alleviates view-specific box-center bias through complementary observations. However, this benefit depends on sufficient viewpoint diversity and on the availability of multiple sufficiently visible observations. When the target is severely occluded in all views, or when all detections exhibit a similar systematic bias, the estimated coarse anchor may deviate beyond the tolerance of the spherical proposal. Future work will investigate more robust geometric constraints, including depth-aware center estimation, weighted ray triangulation, projection-consistency filtering, ellipsoidal proposals, and multi-view voting, to improve object extraction accuracy under challenging conditions. Although the component interfaces are not tied to a particular software implementation, all quantitative results are obtained using one fixed detector, tracker, and depth estimator. The effect of substituting these components remains to be evaluated in future work. The current evaluation is limited by the small intersection between the COCO detector taxonomy and 3D reconstruction datasets that provide multi-view observations together with object-level reference geometry. Accordingly, the reported experiments demonstrate cross-dataset applicability under representative tested conditions rather than broad category-level generalization. Extending the evaluation to additional object categories and scenes remains future work. The present experiments evaluate object-level point cloud extraction from sparse multi-view observations and do not evaluate an end-to-end SLAM system. In particular, online camera tracking, loop closure, map optimization, and long-term map consistency are outside the scope of this study.

Overall, the experimental results suggest that MOT-derived Track-ID association can provide useful temporally or cross-view consistent object cues for object-centric 3D reconstruction. The proposed pipeline may serve as a component of future robotic perception and object-centric mapping or SLAM systems. However, integration into and evaluation within a complete SLAM pipeline remain subjects for future work.

## Figures and Tables

**Table 1:** Object-level extraction results from a fixed DA3-generated scene point cloud on DTU dataset sequence 30 and 105.

**Sequence 30**					
	**CD (mm)↓**	**F-Score@ 5 mm↑**	**Precision@ 5 mm↑**	**Recall@ 5 mm↑**	**Overall↓**
DA3 Raw Scene Cloud	90.97	0.4601	0.2998	0.9882	2.055
**Ours**	12.52	0.9289	0.8776	0.9866	1.285
**Sequence 105**					
	**CD (mm)↓**	**F-Score@ 5 mm↑**	**Precision@ 5 mm↑**	**Recall@ 5 mm↑**	**Overall↓**
DA3 Raw Scene Cloud	15.31	0.8468	0.7629	0.9514	1.848
**Ours**	6.92	0.9057	0.8666	0.9485	1.600

**Table 2:** Results on MVImgNet dataset toy bear category.

	CD (m)↓	F-Score@1%↑	Precision@1%↑	Recall@1%↑
DA3 Raw Scene Cloud	1.23	0.6457	0.4777	0.9959
**Ours**	0.46	0.9323	0.9337	0.9310

**Table 3:** Ablation study of proposed object-level extraction stages on a fixed DA3-generated scene point cloud on DTU dataset sequence 30 and 105.

**Sequence 30**					
	**CD (mm)**↓	**F-Score@ 5 mm**↑	**Precision@ 5 mm**↑	**Recall@ 5 mm**↑	**Overall**↓
DA3 Raw Scene Cloud	90.97	0.4601	0.2998	0.9882	2.055
MOT-Guided Object Extraction	27.22	0.8738	0.7831	0.9882	1.387
MOT + Denoising	12.52	0.9289	0.8776	0.9866	1.285
**Sequence 105**					
	**CD (mm)**↓	**F-Score@ 5 mm**↑	**Precision@ 5 mm**↑	**Recall@ 5 mm**↑	**Overall**↓
DA3 Raw Scene Cloud	15.31	0.8468	0.7629	0.9514	1.848
MOT-Guided Object Extraction	7.31	0.8999	0.8539	0.9513	1.615
MOT + Denoising	6.92	0.9057	0.8666	0.9485	1.600

**Table 4:** One-factor-at-a-time sensitivity analysis on DTU dataset sequence 30 and 105.

**Sequence 30**					
	**CD (mm)↓**	**F-Score@ 5 mm↑**	**Precision@ 5 mm↑**	**Recall@ 5 mm↑**	**Overall↓**
β=0.5	11.535	0.9353	0.8891	0.9865	1.288
β=1.0	12.519	0.9289	0.8776	0.9866	1.285
β=1.5	12.906	0.9256	0.8723	0.9858	1.285
τ=2.5 m	13.045	0.9249	0.8702	0.9870	1.280
τ=5.0 m	12.519	0.9289	0.8776	0.9866	1.285
τ=10.0 m	11.648	0.9346	0.8876	0.9868	1.287
γ=0.25	12.519	0.9289	0.8776	0.9866	1.285
γ=0.5	12.519	0.9289	0.8776	0.9866	1.285
γ=1.0	12.519	0.9289	0.8776	0.9866	1.285
**Sequence 105**					
	**CD (mm)↓**	**F-Score@ 5 mm↑**	**Precision@ 5 mm↑**	**Recall@ 5 mm↑**	**Overall↓**
β=0.5	6.489	0.9094	0.8736	0.9482	1.586
β=1.0	6.921	0.9057	0.8666	0.9485	1.600
β=1.5	7.136	0.9025	0.8606	0.9487	1.604
τ=2.5 m	7.399	0.9011	0.8582	0.9486	1.614
τ=5.0 m	6.921	0.9057	0.8666	0.9485	1.600
τ=10.0 m	6.518	0.9089	0.8730	0.9478	1.588
γ=0.25	6.924	0.9058	0.8665	0.9488	1.600
γ=0.5	6.921	0.9057	0.8666	0.9485	1.600
γ=1.0	6.920	0.9057	0.8666	0.9485	1.600

**Table 5:** Results on ScanNet++ dataset.

	CD (m)↓	F-Score@1%↑	Precision@1%↑	Recall@1%↑	Automatic Cross-View Association
Segmentation Baseline	0.028	0.5315	0.5698	0.4981	✕
MOT + Denoising	0.027	0.5188	0.5626	0.4812	✓

**Table 6:** Runtime study results comparing to segmentation baseline.

**DTU Dataset Sequence 30**				
	**CD (mm)↓**	**F-Score@ 5 mm↑**	**Overall↓**	**Runtime (s)↓**
Segmentation Baseline	5.41	0.9831	1.190	0.3659
MOT + Denoising	12.52	0.9289	1.285	0.3365
**DTU Dataset Sequence 105**				
	**CD (mm)↓**	**F-Score@ 5 mm↑**	**Overall↓**	**Runtime (s)↓**
Segmentation Baseline	3.48	0.9539	1.474	0.3708
MOT + Denoising	6.92	0.9057	1.600	0.3405
**MVImgNet Toy Bear Category**				
	**CD (m)↓**	**F-Score@1%↑**	**Runtime (s)↓**	
Segmentation Baseline	0.46	0.9242	0.3907	
MOT + Denoising	0.46	0.9323	0.3569	
**ScanNet++**				
	**CD (m)↓**	**F-Score@1%↑**	**Runtime (s)↓**	
Segmentation Baseline	0.028	0.5315	0.3722	
MOT + Denoising	0.027	0.5188	0.3254	

Note: Runtime excludes depth prediction and scene-level point cloud construction for both methods. The proposed-method runtime includes detection, appearance-feature extraction, MOT association, geometric proposal, and density refinement. The segmentation-baseline runtime includes segmentation inference, mask projection, and point selection. Manual cross-view association of the ScanNet++ segmentation masks is excluded; consequently, the reported segmentation runtime is a lower-bound estimate for a complete track-consistent pipeline.

## Data Availability

Data is contained within the article.
